# Effective endoscopic submucosal dissection using tented elevation with dental floss traction for a large colorectal laterally spreading tumor with submucosal fibrosis

**DOI:** 10.1055/a-2564-0653

**Published:** 2025-05-06

**Authors:** Jianjiao Lin, Baohua Luo, Tao Su, Chunlin Chen, Yan Liu, Jianxiang Hong, Li Xiang

**Affiliations:** 1 635621 Gastroenterology, The Second Affiliated Hospital of The Chinese University of Hong Kong – Shenzhen, Shenzhen, China; 2 649886 Southern University of Science and Technology Hospital, Shenzhen, China

Large colorectal laterally spreading tumors (LSTs) with submucosal fibrosis present significant challenges for endoscopic resection. Various methods have been proposed to shorten the procedure time for such fibrotic lesions 1 2 . This report highlights the safety and efficacy of endoscopic submucosal dissection (ESD) using a novel traction technique for large colorectal LSTs with severe fibrosis.

A 61-year-old man was hospitalized with a recurrent lateral rectal tumor. The patient had undergone surgical resection for a rectal neoplasm at another institution a year previously; he subsequently developed a more extensive fibrotic tumor over 12 cm in diameter ( Fig. 1 ). Endoscopic ultrasonography (EUS) confirmed the lesion’s origin as being mucosal and muscularis mucosa thickening, with marked submucosal fibrosis ( Fig. 2 ). Computed tomography  revealed a rectal mass with no lymph node or distant organ metastasis.

**Fig. 1:**
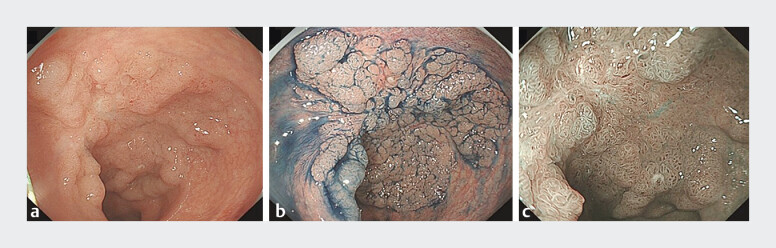
Colonoscopic views showing: **a** a lateral rectal neoplastic lesion measuring approximately 12 cm in diameter; **b** a granular, homogeneous surface with well-defined mucosal boundaries on indigo carmine staining; **c** a well-organized, intricate pit-like branching structure and uniformly thick microvessels on narrow-band imaging (NBI).

**Fig. 2:**
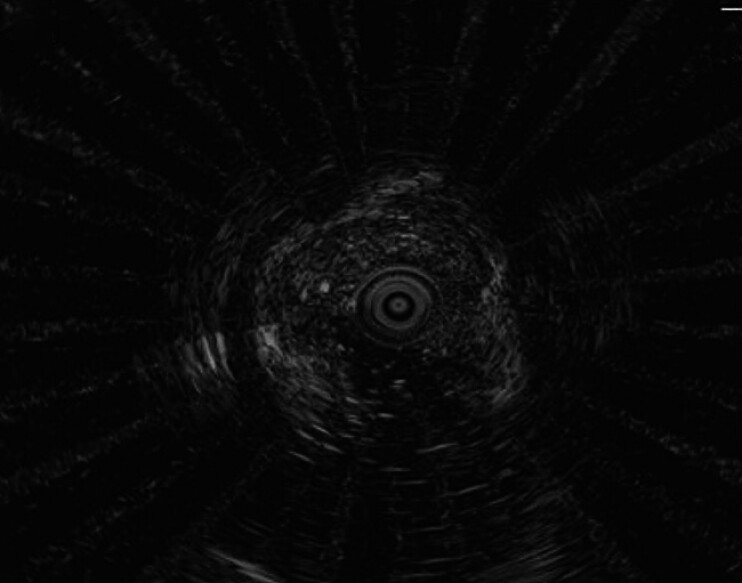
Image during endoscopic ultrasonography showing a slightly hyperintense mass, measuring approximately 122 × 6 mm, originating from the mucosa and muscularis mucosa, along with localized submucosal fibrous hyperplasia.

After the treatment options had been discussed with him, the patient opted for ESD, which was performed successfully ( Video 1 ). The primary challenge was to navigate the submucosal fibrosis while ensuring complete resection of the large lesion. Therefore, tented elevation with dental floss traction was used to allow safe and efficient removal without perforation ( Fig. 3 and Fig. 4 ). The procedure was completed in approximately 2 hours, with the operative time significantly reduced by the use of traction.

A large colorectal laterally spreading tumor with severe submucosal fibrosis was resected safely and efficiently using the tented elevation with numerous tractions (TENT) technique to enable the dissection line to be visualized.Video 1

**Fig. 3:**
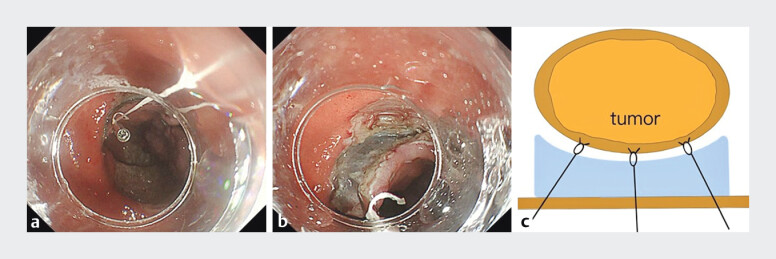
The tented elevation with numerous tractions (TENT) technique: **a, b** is seen on colonoscopic images showing: **a** multiple dental floss traction devices deployed at various points of the dissected specimen and attached to different areas of the contralateral mucosa; **b** the perpendicular alignment of the endoscopic submucosal dissection knife against the submucosal layer after application of the TENT technique; **c** is shown on a schematic diagram.

**Fig. 4:**
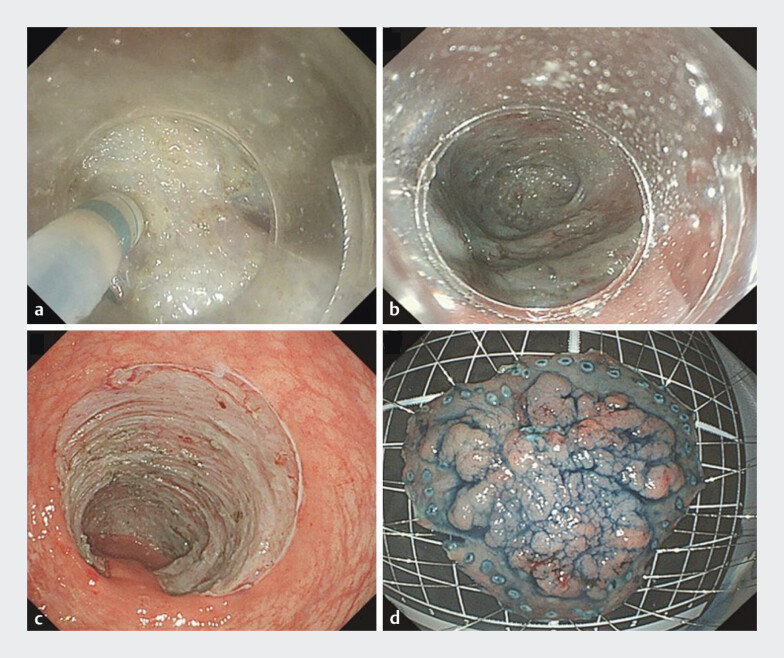
Endoscopic images of the submucosal dissection in an area of severe fibrosis showing: **a** severe fibrosis, with a whitish submucosal layer resembling muscular tissue; **b** the dissection line visualized using the tented elevation traction technique with dental floss; **c** the postoperative defect with an intact muscularis propria and no evidence of perforation; **d** the resected en bloc specimen.

The patient was discharged 72 hours after the procedure, with no complications occurring during follow-up. Histopathology revealed a rectal villous tubular adenoma with high grade intraepithelial neoplasia, without basal invasion and with clear resection margins.

The dental floss traction technique, commonly used in upper gastrointestinal endoscopic resections 3 , was adapted for this case with a multipoint approach, creating a tent-shaped structure for enhanced visualization and resection of the large fibrotic rectal lesion. This method improved efficiency and ensured histologically complete removal, ensuring negative vertical margins, and making it particularly valuable for rectal lesions larger than 10 cm.

Endoscopy_UCTN_Code_TTT_1AQ_2AD_3AD

## Notes

Endoscopy E-Videos https://eref.thieme.de/e-videos
*E-Videos* is an open access online section of the journal *Endoscopy* , reporting on interesting cases and new techniques in gastroenterological endoscopy. All papers include a high-quality video and are published with a Creative Commons CC-BY license. Endoscopy E-Videos qualify for HINARI discounts and waivers and eligibility is automatically checked during the submission process. We grant 100% waivers to articles whose corresponding authors are based in Group A countries and 50% waivers to those who are based in Group B countries as classified by Research4Life (see: https://www.research4life.org/access/eligibility/ ). This section has its own submission website at https://mc.manuscriptcentral.com/e-videos .
